# Supplementary figures and images for: GenArk: Towards a million UCSC genome browsers
Source: Res Sq. 2023 Apr 3:rs.3.rs-2697398. Preprint. [Version 1] doi: 10.21203/rs.3.rs-2697398/v1 (PMC10104252; doi:10.21203/rs.3.rs-2697398/v1)

## Supplementary figures:

### 1. growth of completed assemblies

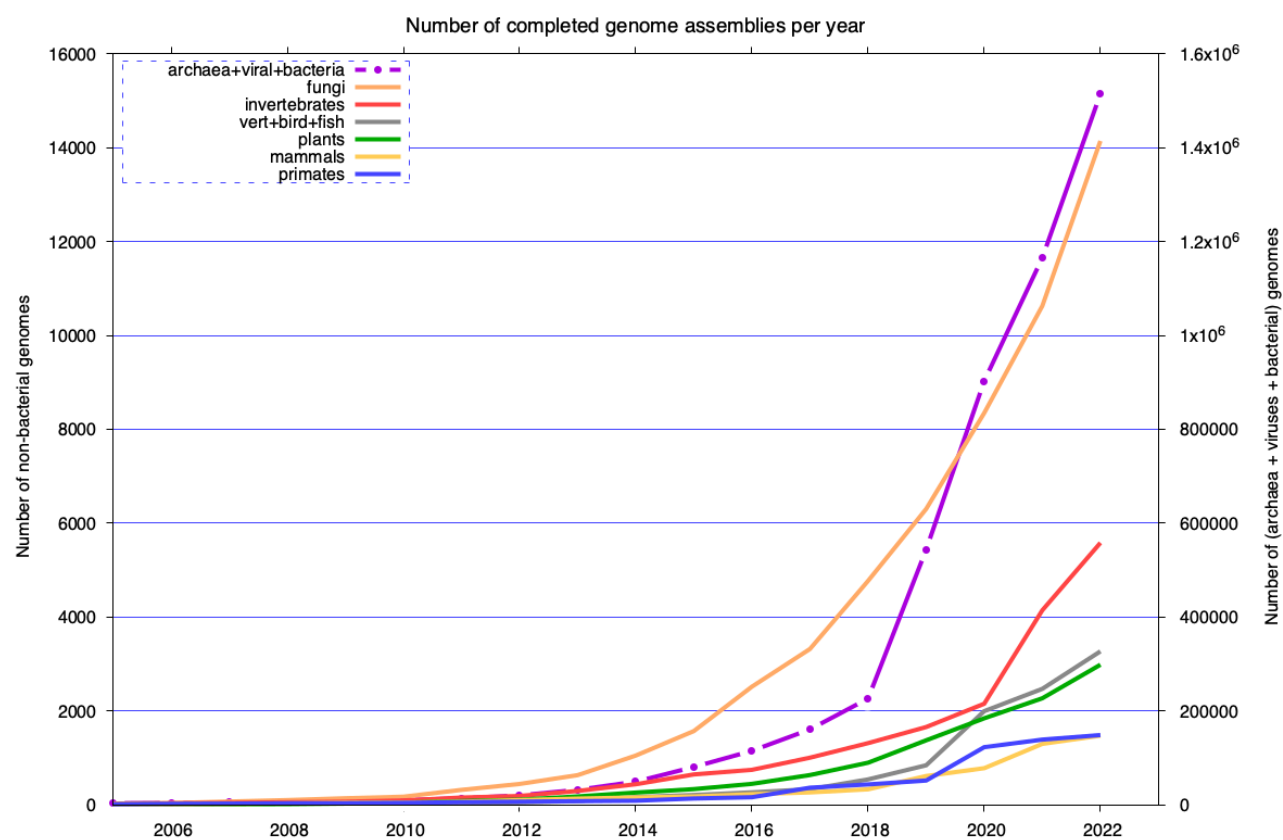

Supplement: 1 [file NIHPPRS2697398V1-supplement-1.pdf]
